# Seasonal changes in daily temperature fluctuation control flowering through a time-dependent regulation of *FLOWERING LOCUS T* in *Arabidopsis*

**DOI:** 10.21203/rs.3.rs-7234857/v1

**Published:** 2025-07-31

**Authors:** Akane Kubota, Yoshinori Kondo, Hiroshi Takagi, Tomoaki Muranaka, Nobutoshi Yamaguchi, Shigeo S. Sugano, Atsushi J. Nagano, Motomu Endo, Takato Imaizumi

**Affiliations:** 1 Division of Biological Science, Nara Institute of Science and Technology, Nara, 630-0192, Japan.; 2 Department of Biology, University of Washington, Seattle, WA, 98195, USA.; 3 Precursory Research for Embryonic Science and Technology, Japan Science and Technology Agency, Kawaguchi-shi, Japan; 4 Bioscience and Biotechnology Center, Nagoya University, Nagoya, Aichi, 464-8601, Japan; 5 Institute for Advanced Research (IAR), Nagoya University, Nagoya, 464-8601, Japan; 6 Graduate School of Bioagricultural Sciences, Nagoya University, Aichi, 464-8601 Japan; 7 The Biomanufacturing and Process Research Center, National Institute of Advanced Industrial Science and Technology, Ibaraki, 305-8566, Japan; 8 Institute for Advanced Biosciences, Keio University, Tsuruoka, Yamagata 997-0052, Japan; 9 Bioscience and Biotechnology Center, Nagoya University, Nagoya, Aichi, 464-8601, Japan

## Abstract

Temperature changes along seasonal transitions control flowering. Under natural long-day conditions, flowering is controlled by a bimodal expression pattern of the florigen gene, *FLOWERING LOCUS T* (*FT*), in *Arabidopsis*. Although cool ambient temperatures delay flowering through *FT* repression, it is unknown how daily temperature fluctuations regulate bimodal *FT* profiles. Seasonal increases in nighttime temperatures are less variable than those during the daytime in spring, potentially providing more reliable timing information. By simulating daily temperature fluctuations of spring, we showed that cool night-to-morning temperatures activate multiple mechanisms to control morning and evening *FT* peaks separately. Lowering night temperatures regulates the CONSTANS (CO) protein function by reducing its stability from midday to dusk and increasing its interaction with a colder-night induced repressor, *B-BOX DOMAIN PROTEIN 29* (*BBX29*), in the morning. Our results demonstrated the dynamics of time-specific regulatory mechanisms in temperature signaling during spring when cooler temperatures determine the timing of flowering.

## Introduction:

Plants monitor surrounding environmental changes and anticipate seasonal transitions by adjusting their developmental patterns to maximize their fitness. Transition to reproductive growth (i.e., flowering) is regulated by light (photoperiod) and temperature, two major environmental factors that not only change daily but also convey seasonal information^1,2^. In *Arabidopsis*, when the day length becomes long enough to induce flowering, a transcription factor, CONSTANS (CO), directly induces the *FT* expression around dusk to promote flowering^2–5^. While the circadian clock regulates the diurnal expression of *CO*, light and temperature signals regulate the stability of CO protein through multiple RING-type E3 ubiquitin ligases, thereby forming a hub that integrates photoperiod and temperature cues^6–9^. Actions of these factors on *FT* regulation around dusk have been extensively studied by using conventional laboratory long-day conditions (i.e., constant 22°C and 16-hour light/8-hour dark conditions).

However, natural long-day conditions lead to *FT* induction both in the morning and at dusk, resulting in earlier flowering compared to conventional laboratory long-day conditions^10^. Similar bimodal expression patterns of *FT* homologs are widely observed in long-day plants including crops^11–15^, suggesting that time-of-day dependent regulation of *FT* might be conserved across long-day plant species. Induction of morning *FT* requires long-day photoperiod along with Far-Red (FR) light supplementation that resembles the red (R)-to-FR ratio of natural sunlight^10,16^. While FR signals activate *FT* expression, cool ambient temperature repress it through multiple pathways^10^.

Current understandings of temperature-regulated flowering mechanisms mostly rely on the results from the comparison of constant high- or low-temperature regimes throughout a day, while under natural environments, temperature fluctuates drastically on both seasonal and daily timescales. Previous studies have shown that temperature fluctuation alters flowering responses compared to constant temperature conditions, across a variety of thermal ranges^17–19^. In barley, even a small 2°C day-night temperature difference delayed flowering time by up to seventy days compared to constant temperature^20,21^. In *Arabidopsis*, the nighttime temperature has a dominant effect on the regulation of *FT* expression at dusk^22,23^, with mathematical models also supporting its significance in predicting flowering time under autumn-to-winter field conditions^24^. These findings across the species highlights the importance of daily temperature fluctuation to understand flowering regulation. Moreover, temperature signaling is gated differently by the circadian clock in a time-of-day-dependent manner^25^, suggesting that not only the amplitude but also the timing of temperature changes critically influences developmental outcomes. This implies a limitation in extrapolating results obtained from constant-temperature conditions to natural environments.

Our previous study further demonstrated that daily temperature oscillation, but not changes in light intensity, altered the expression pattern of *FT* and flowering time under long-day conditions^10^. Specifically, we recreated bimodal *FT* expression in laboratory settings by applying gradual temperature transitions that mimicked the average temperature traces around the summer solstice in Seattle (47 degrees north: °N), where day length and daytime average temperature resembles conventional laboratory long-day conditions^10^. Although this approach was effective, it involved over a dozen incremental temperature transitions, making it impractical for dissecting time-specific regulatory mechanisms. A recent study described one mechanism by which temperature fluctuation affects morning *FT* expression^26^, but how plants decode daily temperature fluctuation to precisely regulate *FT* expression remains elusive. To overcome this limitation, we developed a simplified two-step temperature regime that preserves the essential features of natural thermoperiods, namely daily mean, amplitude, and timing of temperature transition. This approach allowed us to understand how plants interpret time-sensitive temperature cues to regulate flowering.

Building upon this approach, we characterized springtime temperature trends and established a simplified two-step temperature regime that still recapitulates both bimodal *FT* expression and flowering time observed under natural long-day conditions. We further demonstrate that the night-to-morning temperature serves as a regulatory window, coordinating two distinct temperature-responsive mechanisms that modulate bimodal *FT* expression profiles.

## Results:

### A simplified daily temperature scheme enabled the analysis of the impacts of temperature fluctuation on flowering responses

Our previous observation showed that wild-type (Col-0) plants grown in April took substantially longer to flower than those grown in May outside in Seattle^10^. Photoperiods were already long in these months (approximately 14 hours in April vs. 15 hours in May in Seattle), and this one-hour difference in photoperiods is not likely to account for the observed flowering difference^16^. We therefore hypothesized that seasonal temperature differences may play a dominant role in regulating flowering time during these months.

**Fig.1:**
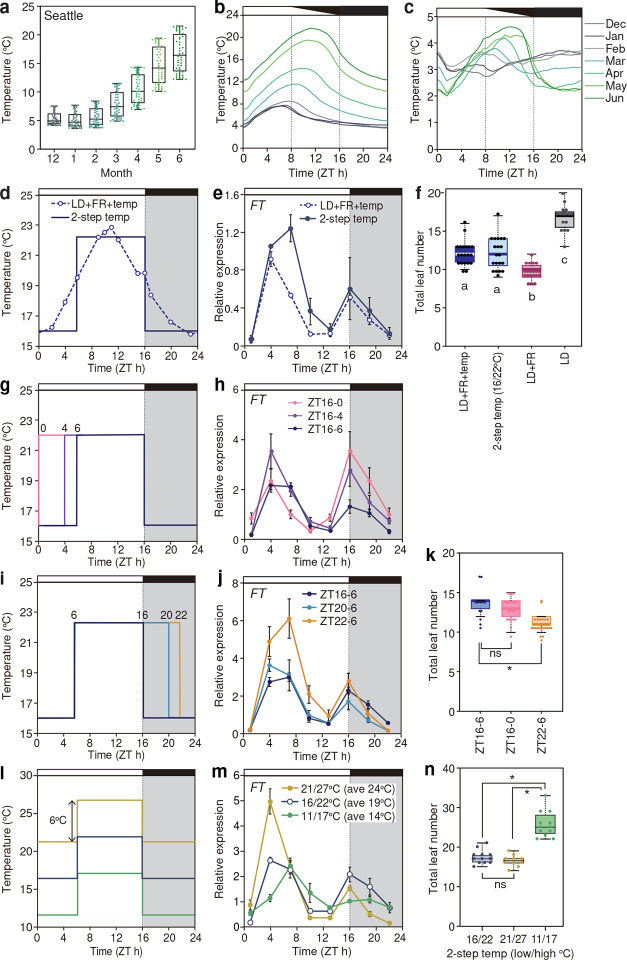
Temperature changes at a specific time of the day differently influence bimodal *FT* expression patterns in long days. **a-c,** Temperature records from 2012 to 2019 in Seattle. Monthly (**a**) and daily trends of average temperatures (**b**), and standard deviations of daily temperatures (**c**) are shown. Each dots in (**a**) represents average temperature from every 30 minutes. The sunrise time was set as Zeitgeber time 0 (ZT0). **d,** Comparison of temperature settings of experimental LD conditions. White/black rectangles and gray shading in the graph represent light/dark periods. **e,** Expression profiles of *FT* under the conditions shown in (**d**). The geometric mean value from gradual temperature conditions was set to 1. All gene expression results (means ± s.e.m.) in this paper were normalized against *IPP2* and *PP2A* from three biologically independent samples. **f,** Flowering time from WT plants grown under experimental LD conditions with different settings of temperature and light conditions. For all the boxplot graphs in this paper, each box is located between the upper and the lower quartiles and the whiskers indicate the 1.5-times interquartile ranges. The horizontal lines in the boxes represent the median. Letters indicate significantly different groups (*p* < 0.05) based on Tukey’s HSD test. 16 ≦ *n* ≦ 20. **g-n**, Effects of timing differences in temperature shifts (**g**-**k**), or average temperatures (**l**-**n**) on *FT* expression. Expression profiles of *FT* (**h**, **j**, **m**) and flowering time results (**k**, **n**) obtained under the corresponding conditions (**g**, **i**, **l**) were shown by the same color. *FT* expression values are relative to the geometric mean values from 16/22°C, ZT6-16 (as indicated in the solid blue line with filled circles). For flowering time analyses, asterisks indicate significantly different groups (*p* < 0.05) and ns indicates no significance based on Bonfferoni-corrected Welch’s T-test (*n* = 20, **k**) or Tukey’s HSD test (10 ≦ *n* ≦12, **n**). ± s. e. m.

To understand how much extent daily temperature fluctuations contribute to seasonal variation in flowering time in spring, we first analyzed temperature trends from winter to *Arabidopsis* growth seasons (March to June) in Seattle and also at different locations in Europe (ranging from 40°N to 60°N), where *Arabidopsis* originated (Fig. 1a–c, Supplementary Fig. 1). From winter to spring, mean value and amplitude of monthly temperatures increased and temperature differences between day and night became larger (Fig. 1a). In addition, comparison of monthly average temperature revealed that there is a seasonal trend in daily temperature fluctuations. During the winter months (December to February), daily temperature patterns resembled, and the changes occurred mainly in the early half of the day (Fig. 1b). In contrast, spring (March to June) showed monthly divergence in temperature profiles and expanding temperature ranges (Fig. 1b). We next analyzed the temperature variability in each month by calculating standard deviations of daily oscillations. Interestingly, the standard deviations during winter were consistent throughout the day (Fig. 1c), while in spring, temperature from night-to-morning window showed lower standard deviations than those of mid-day temperature (Fig. 1c). This indicates that night-to-morning temperatures in the spring season show more predictable incremental changes during those months. This observation led us infer that night-to-morning temperature could serve as a reliable indicator of seasonal progression.

To address the precise time-dependent effect of temperature on bimodal *FT* expression and flowering responses, we simplified our temperature conditions from gradual changes into two-step binary changes. Under long-day conditions where R-to-FR ratio was adjusted to resemble natural sunlight throughout the light period, we changed temperatures in the midpoints of ascending (at Zeitgeber Time 6, ZT6) and descending temperature settings (at ZT16) between high (22°C) and low (16°C) (Fig. 1d, Supplementary Fig. 2a-c^26^). We found that the two-step temperature changes (designated as “2-step temp”) reproduced *FT* expression patterns and flowering time equivalent to those under gradual temperature changes (designated as “LD+FR+temp”) (Fig. 1e, f). In addition, expression patterns of key flowering regulators such as *CO* and *TSF* were similar between the 2-step temp and the gradual temperature changes (Supplementary Fig. 2d, e). *FT* expression at each time point closely matched between 2-step temp and gradual temp conditions (Supplementary Fig. 2f, g), but differed significantly from the constant 22°C condition (LD+FR) (Supplementary Fig. 2h, i), underscoring the regulatory impact of temperature fluctuation. Importantly, three-day treatment of 2-step temp conditions was sufficient to mimic flowering gene expression patterns observed in two-week treatment (Supplementary Fig. S2 j-l), minimizing growth interference while allowing systematic testing.

To examine the effect of temperature fluctuations on flowering response, we focused on three aspects: the timing of temperature transitions, average temperature, and the amplitude of fluctuation, using *FT* expression pattern as a molecular readout. When the low-to-high transitions (normally occurring around dawn) were shifted from ZT0 to ZT6, the evening *FT* peak was markedly reduced, whereas morning *FT* peak remained largely unaffected (Fig. 1g, h, Supplementary Fig. 3a, b). Conversely, altering the timings of high-to-low temperature transitions around dusk from ZT16 to ZT22 (denoted ZT6–22) increased the levels of the morning *FT* without changing the evening peak (Fig. 1i, j, Supplementary Fig. 3c, d). WT plants grown under the ZT6–22 conditions flowered slightly earlier than those under the ZT6–16 conditions (Fig. 1k), indicating the timing of temperature transitions also influences flowering. Expression patterns of *CO* and *TSF* were also altered in accordance with temperature shifts, as is reported for *CO* induction by cool temperature^23^(Supplementary Fig. 3e-h^23^). Taken together, our results indicate that the timing of temperature transition affects *FT* expression patterns in a time-dependent manner, which in part affects flowering time.

Next, we analyzed the effect of average temperature under a fixed 6°C day-night amplitude (Fig. 1l, m). When the average temperature was increased from 14°C (i.e. 11/17°C) to 24°C (i.e. 21/27°C), *FT* expression levels during daytime were gradually increased, with flowering acceleration notably between 14°C and 19°C (Fig 1m, n). These results suggest that temperature rise enhances *FT* expression and flowering. Interestingly, *FT* expression profiles and flowering time varied even when average temperatures were matched, depending on the specific combination of daytime and nighttime temperature (Supplementary fig. 3i-k). These findings revealed that the timings of temperature transitions around dawn and dusk differentially affect morning and evening *FT* expression. Means and amplitudes of daily temperature changes also influence *FT* profiles. Thus, our results indicate that the time-of-day information is crucial to study the impacts of temperature on *FT* patterns and flowering.

### Cooler temperatures destabilize CO protein and repress *FT* expression towards dusk

**Fig. 2:**
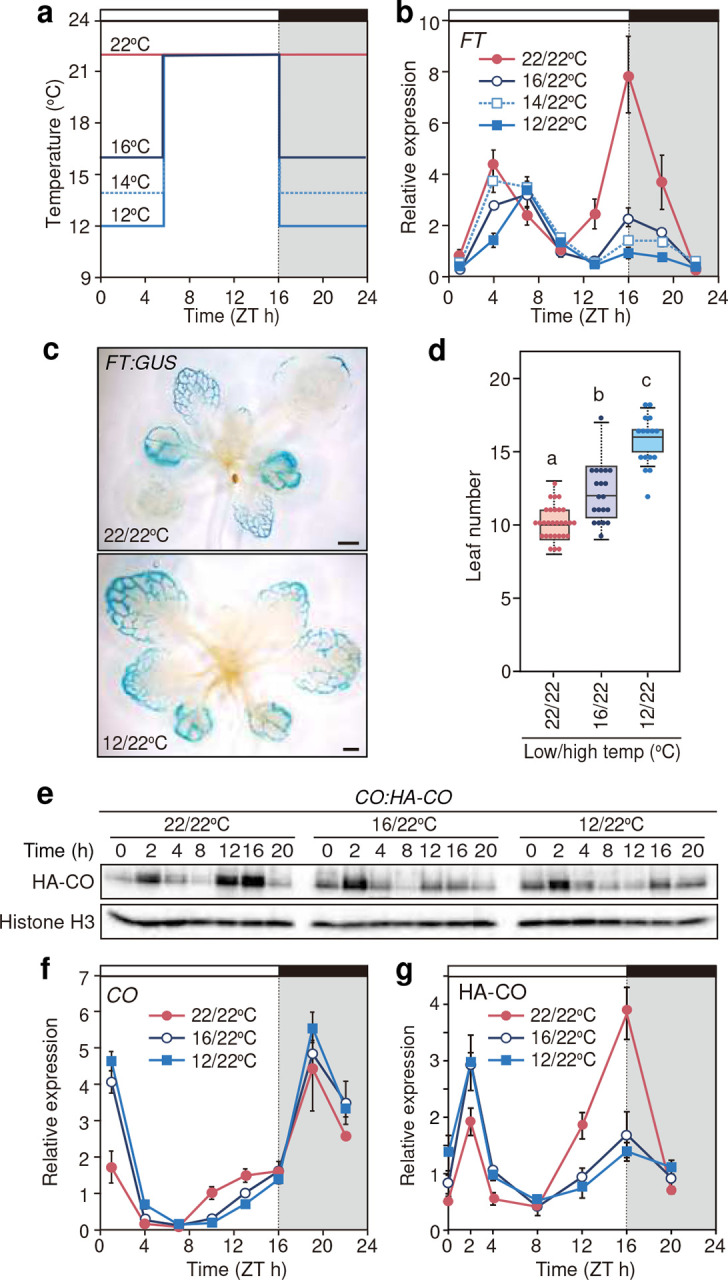
Reduction in night-to-morning temperature destabilizes CO protein around dusk and represses evening *FT* expression **a**, Comparison of the 2-step temperature conditions with different night-to-morning temperatures. **b,** Expression profiles of *FT* under the conditions shown in (**a**). **c,** The spatial expression patterns of *FT* analyzed by GUS staining of the *FT:GUS* plants harvested at ZT4. **d,** Flowering time results of WT under the conditions described in (**a**). Each box is located between the upper and the lower quartiles and the whiskers indicate the 1.5-times interquartile ranges. The horizontal lines in the boxes represent the median. Letters indicate significantly different groups (*p* < 0.05) based on Tukey’s HSD test. 16 ≦ *n* ≦ 29. **e**, A representative western blot image of HA-CO protein present in nuclear-enriched fractions derived from the *CO:HA-CO* plants grown under three temperature conditions shown in (**a**). Histone H3A signal is shown as a loading control. **f**, Expression profiles of *CO* transcripts in WT plants. **g**, Quantification of HA-CO protein signals relative to those of Histone H3A. Data (means ± s.e.m., *n* = 3) were normalized against the geometric mean values obtained from 22/22°C. Plants were grown under 2-step temperature conditions with different night-to-morning temperatures as shown in (**a**).

As we hypothesized that plants monitor night-to-morning temperature to assess seasonal advances in early spring, we analyzed how changes in the night-to-morning temperature influence *FT* expression patterns and flowering. We grew plants in long-day conditions with different low temperatures from ZT16 to the next morning (by ZT6) (Fig. 2a). Reduction in night-to-morning temperatures more pronouncedly suppressed the levels of evening *FT* expression (Fig. 2b). Cooler night-to-morning temperatures also delayed the peak timings of morning *FT* up to 3–4 hours, while the peak timing of the evening *FT* remained the same (Fig. 2b). These results imply that morning and evening *FT* may be regulated by different temperature-controlled mechanisms in a time-dependent manner. We also analyzed the impacts of these temperature changes on the spatial expression patterns of *FT* and found that these temperature conditions did not obviously alter the vascular-specific expression patterns of *FT:GUS* (Fig. 2c). In addition, night-to-morning temperature reduction gradually delayed flowering time in WT, possibly due to the reduction of *FT* expression levels (Fig. 2d). Involvement of *FT* in the temperature-dependent flowering regulation is supported by our observation that *ft* mutants (*ft-101* and *ft-1*) and the *co* mutant showed late-flowering phenotypes compared to WT (Supplementary Fig. 4). In addition, the *ft-1 tsf-1* double mutants flowered later than the *ft-1* mutant under both temperature conditions (Supplementary Fig. 4a), suggesting the additive effect of *FT* and *TSF* in the regulation of flowering time. However, all these mutants flowered slightly later under 12/22°C than 22/22°C, indicating that additional floral regulators, such as *SUPPRESSOR OF OVEREXPRESSION OF CONSTANS 1* (*SOC1*), or *SHORT VEGETATIVE PHASE* (*SVP*), may also contribute to temperature-mediated flowering regulation^27–30^. These results suggest that decreases in night-to-morning temperature delay flowering time and that the *CO-FT* pathway is involved in this regulation.

To investigate how cooler temperatures affect the *CO-FT* pathway, we examined the expression patterns of *CO* transcript and CO protein under different temperature conditions (Fig. 2e–g). Compared to the 22/22°C conditions, cool nigh-to-morning temperature induced the transcription of *CO* at dawn while retarding the midday-to-dusk induction, in a similar manner under 16/22°C and 12/22°C (Fig. 2f). Under these conditions, cooler temperatures reduced CO protein accumulation towards the dusk by approximately half, even though the midday temperatures for all conditions were uniformly set to be 22°C (Fig. 2e, g, Supplementary Fig. 4b). These results indicate that the effect of cooler temperatures from nighttime to morning persists even after temperature rise during the day, leading to reducing CO protein stability towards the following dusk, thereby suppressing evening *FT* expression.

### Cooler temperatures induce the expression of *BBX29* and *BBX31* in the morning

**Fig. 3:**
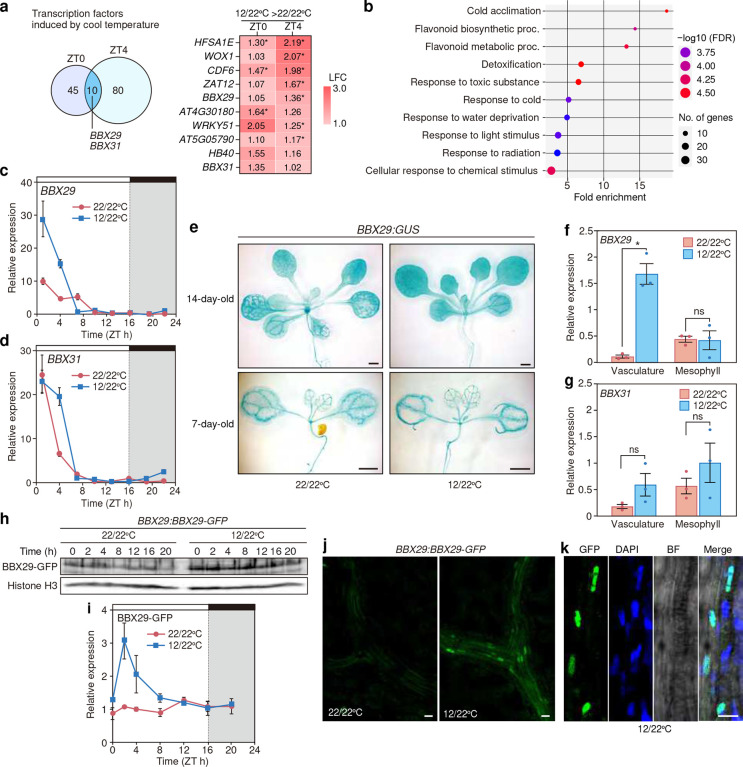
Reduction in night-to-morning temperature induces morning expression of *BBX29* in the vasculature **a**, Venn diagram showing overlap among the transcription factors upregulated under 12/22°C compared to 22/22°C between ZT0 and ZT4. Ten overlapped genes with their expression levels (in Log_2_ fold change: LFC) were listed. **b**, Summary of enriched GO biological process terms among genes upregulated on ZT4 under 12/22°C compared to 22/22°C (*p* < 0.05, Counts per million reads mapped (CPM) >1). **c-d,** Effects of night-to-morning reduction on expression profiles of *BBX29* (**c**) and *BBX31*(**d**). *FT* expression values are relative to the geometric mean value from 22/22°C. **e,** The spatial expression patterns of *BBX29* analyzed by GUS staining of the *BBX29:GUS* plants harvested on ZT1. **f-g,** Expression of *BBX29* and *BBX31* transcript in mesophyll and vasculature tissues isolated from WT plants at ZT1. Bars represent the means, and error bars represent plus/minus s. e. m. (*n* = 3). Asterisks indicate significantly different groups (*p* < 0.05) and ns indicates no significance based on Welch’s T-test or Student’s T-test. Comparison was made between the plants grown under 22/22°C or 12/22°C. **h**, A representative western blot image of BBX29-GFP protein present in nuclear-enriched fractions derived from the *BBX29:BBX29-GFP* plants. Histone H3A signal is shown as a loading control. **i**, Quantification of BBX29-GFP protein amounts relative to those of Histone H3A. Data (means ± s.e.m., *n* = 3) were normalized against the geometric mean values obtained from 22/22°C. **j,** Confocal fluorescent images of the *BBX29:BBX29-GFP* plants showing subcellular localization of BBX29-GFP at ZT4. Leaf blades of *BBX29:BBX29-GFP* plants grown under 22/22°C and 12/22°C were mounted. **k,** Magnified images of vasculature bundles in *BBX29:BBX29-GFP* plants from ZT4 of 12/22°C. The nuclei were stained by DAPI. BF; bright field. Scale bars, 10 μm.

Our results showed that the reduction of evening *FT* by cool night-to-morning temperature can be explained in part by the destabilization of CO towards dusk. While night-to-morning temperature reduction by 12°C also repressed morning *FT* expression levels, CO protein in the morning tend to be stabilized under 12/22°C than that in 22/22°C (Fig 2g, Supplementary Fig. 4b), suggesting that there may be morning-specific *FT* repressors especially around 12°C. To identify such *FT* repressors, we performed RNA sequencing (RNA-seq) experiment in the samples harvested in the morning (ZT0 and ZT4) of 22/22°C and 12/22°C (Fig. 3a). Among genes induced by lower temperature, gene ontology terms such as “water deprivation” or those related to flavonoid metabolism were overrepresented along with cold-related responses (Fig. 3b), suggesting the partial relation between cold temperature responses and ABA signaling^31 32^, or flavonoid metabolic processes^33,34^. Among transcription factors differentially expressed both on dawn (ZT0) and in the morning (ZT4) of 12/22°C, we found that two members of the *BBX* family genes that belong to Group V, namely *BBX29* and *BBX31*, were highly induced under 12/22°C conditions (Fig. 3a, Supplementary Table S1). *A. thaliana* has 32 members of B-box genes that are classified into 5 groups based on the domain structures. While CO (BBX1) belongs to Group I, which consists of two B-box domains and a DNA-binding CCT domain at the C-terminus, BBX26–32 belong to Group V, which contains only single B-box domain^35^. Since both BBX29 and BBX31 (also known as Micro Protein miP1a) are reported to regulate flowering^36–38^, we speculated that these two genes are specifically involved in flowering regulation under cool temperatures. Time-course gene expression analyses revealed that *BBX29* and *BBX31* were highly induced in the morning of 12/22°C (Fig 3c, d, Supplementary Fig. 5a-d), consistent with previous studies showing that some Group V *BBX* genes are cold-responsive genes^39–41^. Detailed time-course analyses of *BBX29* expression showed that cool temperature gradually increased *BBX29* expression two hours before dawn, then the expression level rose approximately four times higher within an hour after light onset (Supplementary Fig. 5e). Rapid induction of *BBX29* after dawn was attenuated in the mutant of morning-phased clock genes, *CIRCADIAN CLOCK ASSOCIATED1* (*CCA1*) and *LATE ELONGATED HYPOCOTYL* (*LHY*) (Supplementary Fig. 5e), indicating the clock regulation of *BBX29* expression. In line with the previous findings that *CCA1* and *LHY*, together with their homologs, *REVEILLE*s, induce Group V *BBX* genes in response to cold^42,43^, our results highlight the importance of the circadian clock in the thermoperiodic responses.

To further investigate if cool temperature induces the expression of *BBX29* in a tissue-specific manner, we performed histochemical staining of *BBX29:GUS* and RT-qPCR analysis using protoplast-based tissue isolation from WT plants under 22/22°C and 12/22°C conditions^44^ (Fig. 3e–g, Supplementary Fig. 5f-k). Stronger staining was observed in vasculatures of the 7-day-old *BBX29:GUS* plants and staining levels of the entire leaves increased in the 14-day-old plants in 22/22°C and 12/22°C conditions, but there was no clear and consistent difference in staining patterns between two temperature conditions (Fig. 3e). However, our tissue-enriched gene expression analysis revealed that cool temperature highly induced *BBX29* transcription in isolated vasculature-enriched samples, but not in mesophyll-enriched ones (Fig. 3f). Cool temperature altered the expression of other Group V members, but the effect was not as strong as *BBX29* (Fig. 3f, Supplementary Fig. 5h-k). Since BBX29 localizes in the nucleus and undergoes COP1-mediated protein degradation in light- and brassinosteroid-dependent manners^45,46^, we examined whether BBX29 is posttranscriptionally regulated in a temperature-dependent manner. Protein accumulation of nuclear-enriched *BBX29:BBX29-GFP* showed that BBX29-GFP protein in the morning is more abundant under 12/22°C compared to 22/22°C (Fig. 3h, i). Furthermore, BBX29-GFP nuclear localization was detected almost exclusively in some vasculature cells in the morning of 12/22°C, but not under 22/22°C (Fig. 3j, k). These results suggest that cool temperature enhances the amount of nuclear-localized BBX29 and/or nuclear-translocation of BBX29 in the vascular tissues.

### BBX29 is a flowering repressor under cool night-to-morning temperature conditions

**Fig. 4:**
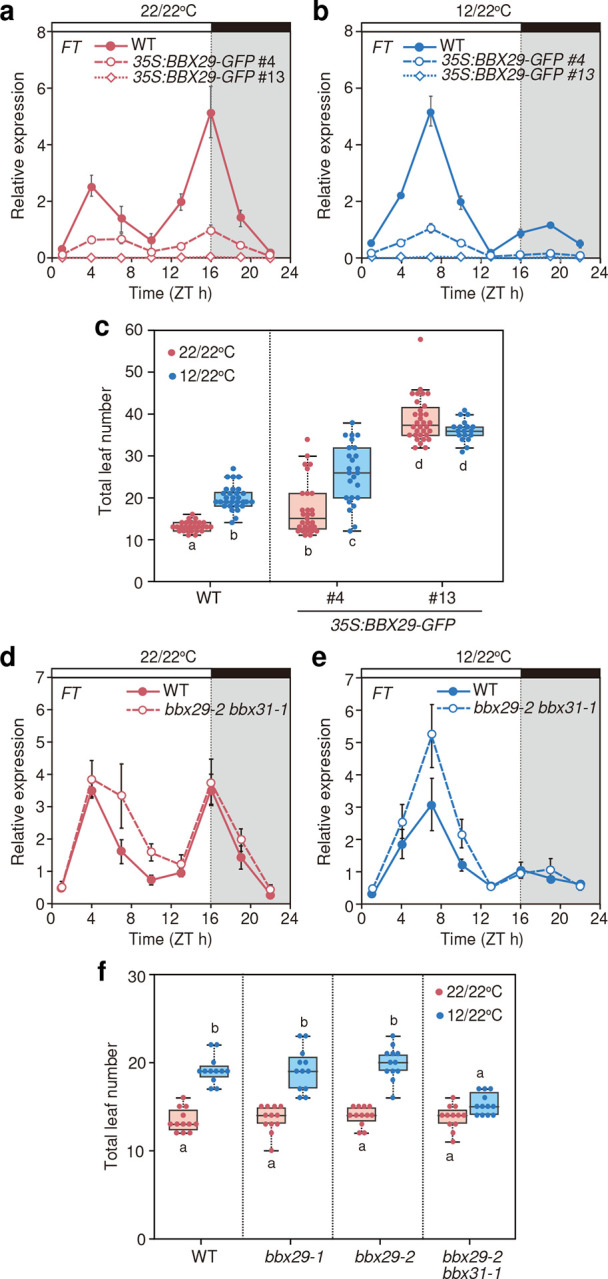
*BBX29* and *BBX31* repress morning *FT* expression in response to cool temperatures happening from night to morning **a-c,** *FT* expression profiles (**a, b**) and flowering time results (**c**) of WT and *35S:BBX29-GFP* plants under 22/22°C (**a**) and 12/22°C (**b**). **d-f**, *FT* expression profiles (**d, e**) and flowering time results (**f**) of WT and *bbx29 bbx31* mutants under 22/22°C (**d**) and 12/22°C (**e**). For flowering experiments (**c, f**), box plots display the upper and the lower quartiles and the whiskers indicate the 1.5-times interquartile ranges. The horizontal lines in the boxes represent the median. Letters indicate significant differences based on Tukey’s HSD test (*p* < 0.05, 27 ≦ *n* ≦ 32 for **c**, *n* = 12 for **f**).

Because cool night-to-morning temperature enhances the accumulation of *BBX29* transcript and protein in the vasculature in the morning, we investigated the function of *BBX29* in the regulation of morning *FT*. Since previous studies have reported both promotive and repressive roles of *BBX29* in flowering regulation^37,38^, we analyzed the specific contribution of *BBX29*, together with *BBX31*, in the regulation of bimodal *FT* expression. We first analyzed the flowering phenotypes of *BBX29-*overexpressors. Under 22/22°C and 12/22°C conditions, overexpression of *BBX29* severely repressed the expression levels of *FT* and *TSF* throughout the day and delayed flowering (Fig. 4a–c, Supplementary Fig. 6a, b, d, e). Overexpression of other Group V *BBX* members, including *BBX31*, also caused late flowering, as reported previously^47–49^ (Supplementary Fig. 7a-f)^47–49^. In the *35S:BBX29-GFP* lines, the expression of *CO* was hardly affected, while the expression of Group V *BBX* members was severely repressed (Supplementary Fig. 6c, f, g-n), potentially due to the negative feedback regulation within the Group V genes^45^.

In contrast to significant *FT* repression in the *35S:BBX29-GFP* lines, *FT* expression was only slightly enhanced in the *bbx29* single mutant (Supplementary Fig. 7g-k). In the double mutant of *bbx29 bbx31*, morning *FT* expression was elevated under 12/22°C, suggesting that *BBX29* and *BBX31* redundantly repress morning *FT* (Fig. 4d, e). Flowering time in the single mutants of *bbx29* was almost identical to those in WT, but *bbx29 bbx31* double mutant flowered significantly earlier than WT under 12/22°C, resulting in nearly comparable flowering time between 12/22°C and 22/22°C (Fig. 4f). These results indicated that *BBX29* and *BBX31* act as flowering repressors through the regulation of morning *FT* expression under long-day conditions with the cool night-to-morning temperature.

### Cool night-to-morning temperature enhances the complex formation between BBX29 and CO to repress *FT* expression

**Fig. 5:**
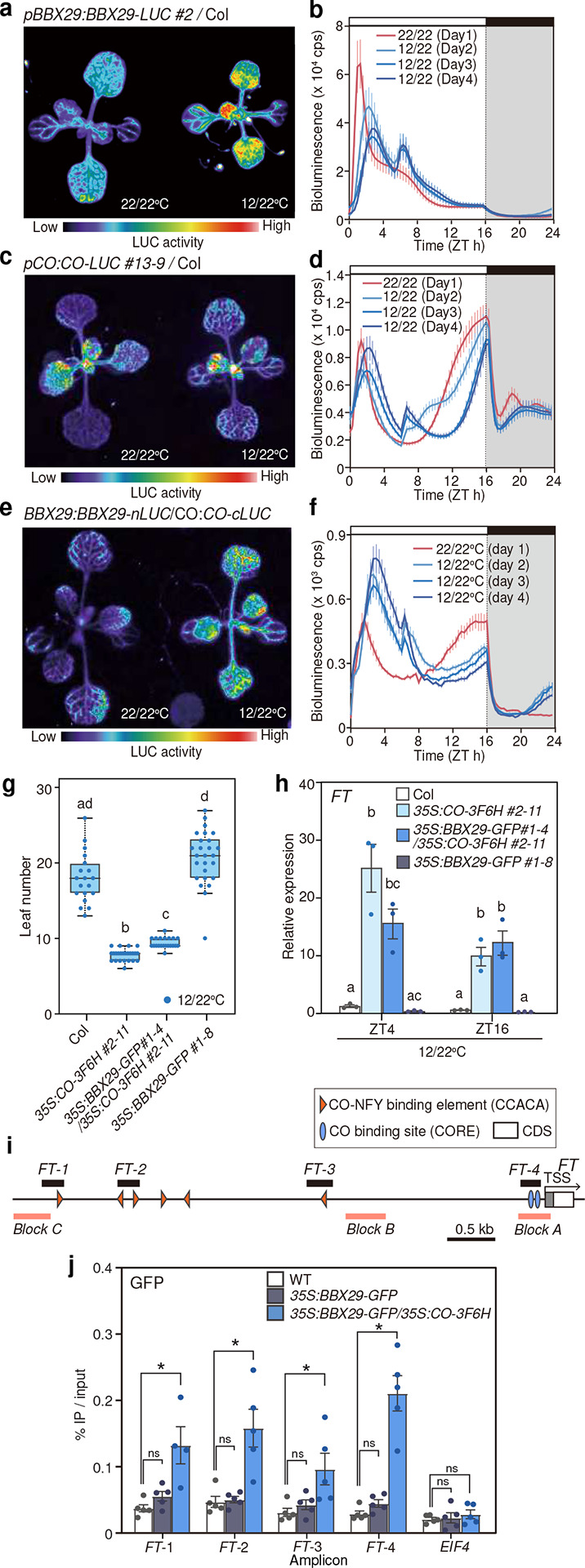
Cool temperature enhances BBX29-CO complex formation in the morning **a-f**, Representative images of bioluminescence from plants harboring *BBX29:BBX29-LUC* (**a**), *CO:CO-LUC* (**c**), and *BBX29:BBX29-nLUC/CO:CO-cLUC* (**e**). Images were taken at ZT2 of 22/22°C and 12/22°C. Graphs represent real-time monitoring of bioluminescence from *BBX29:BBX29-LUC* (**b**), *CO:CO-LUC* (**d**), and *BBX29:BBX29-nLUC/CO:CO-cLUC* (**f**) plants during the transition from 22/22°C (Day 1) to 12/22°C (Day 2 to Day 4). Data are presented in means± s.e.m. (n=10). cps; counts per second. **g-h**, *FT* expression (**g**) and flowering time (**h**) of plants overexpressing *CO-3F6H* and/or *BBX29-GFP* under 12/22°C. The expression values are relative to the mean value of wild-type gene expression under 12/22°C. Box plots display the upper and the lower quartiles and the whiskers indicate the 1.5-times interquartile ranges. The horizontal lines in the boxes represent the median. Letters indicate significant difference based on Tukey HSD test (**h**, *p* < 0.05, 19 ≦ *n* ≦ 35). Bars represent the means, and error bars represent plus/minus s. e. m. Letters indicate significant differences based on Tukey’s HSD test (**g**, *p* < 0.05, *n* = 3). **i**, Schematic diagram of *FT* locus showing the positions of enhancers^52^. The ampicons used for ChIP qPCR and CO-binding regions (CCACA motif and CORE element) are indicated. **j**, ChIP-qPCR results testing the binding of BBX29-GFP on *FT* promoter at ZT3. Bars represent the means, and error bars represent plus/minus s. e. m. (*n* = 5). Asterisks indicate significant differences against WT controls and ns indicates no significance, based on one-tailed Dunnet’s test (*p* < 0.05).

Our results showed that a reduction in night-to-morning temperature induces the transcription of *BBX29*, leading to morning *FT* repression. Given that BBX29 physically interacts with CO (Supplementary Fig. 8a), we decided to monitor protein accumulation and interactions with high spatiotemporal resolution by using *in vivo* live imaging of luciferase (LUC) reporter driven by endogenous promoters. C-terminus LUC fusion of BBX29 and CO driven by their own promoters can complement the flowering phenotype of the mutant, validating their functionalities (Supplementary Fig. 8b-d). When plants expressing *BBX29:BBX29-LUC* were transferred from 22/22°C to 12/22°C, BBX29-LUC showed prolonged accumulation throughout the morning and broadened spatial expression (Fig. 5a, b). Temperature shift from 22/22°C to 12/22°C did not alter vascular-specific expression of CO-LUC, while its degradation during midday and re-accumulation towards dusk were both delayed (Fig. 5c, d). We then tested the protein-protein interaction of BBX29 and CO in the plants harboring *BBX29:BBX29-nLUC/CO:CO-cLUC* (Fig. 5e, f). Under 22/22°C, vascular-specific interaction of BBX29 and CO was observed both in the morning and evening (Fig. 5e, f, Supplementary Fig. 9a-c). Upon the transfer to 12/22°C on ZT0, BBX29-CO interaction was rapidly induced and maintained at the high level during the morning, while the interaction in the evening was reduced by half (Fig. 5e, f, Supplementary Fig. 9a-c). Cool temperature also enhanced BBX29-BBX29 interaction during the morning in a spatiotemporal manner, which may be involved in various photomorphogenic responses^41,46,50,51^ (Supplementary Fig. 9d-h). These results suggest that BBX29 and CO strongly interact in the vasculature during the morning of 12/22°C conditions.

To assess the consequence of BBX29-CO interaction, we introduced *35S:BBX29-GFP* into *35S:CO-3F6H* and observed flowering phenotypes. Expression of *FT* in *35S:CO-3F6H* was repressed by co-expression of *35S:BBX29-GFP* particularly in the morning of 12/22°C conditions, resulting in a later flowering phenotype (Fig. 5g, h; Supplementary Fig. 10a-d). As *CO* transcript levels were not changed in these lines (Supplementary Fig. 10c), our results support the notion that BBX29 modulates *FT* expression through its interaction with CO. To further test whether the reduction of *FT* is caused by the direct binding of BBX29-CO protein complex on *FT* locus, we performed ChIP qPCR with *BBX29-CO* overexpression plants (Fig. 5i, j). While little enrichment of BBX29-GFP was observed in the single overexpression of *BBX29-GFP*, co-overexpression of *BBX29-GFP* and *CO-3F6H* significantly enhance the DNA-binding of BBX29-GFP in both transcriptional starting sites (TSS) where CO-binding elements exist, and distal enhancers required for full spatiotemporal expression of *FT*^52,53^ (Fig. 5i). These results suggest that CO recruits BBX29 to *FT* promoter, thereby downregulating the expression of *FT* in the morning.

### B-box domain of BBX29 plays a critical role in flowering repression

**Fig. 6:**
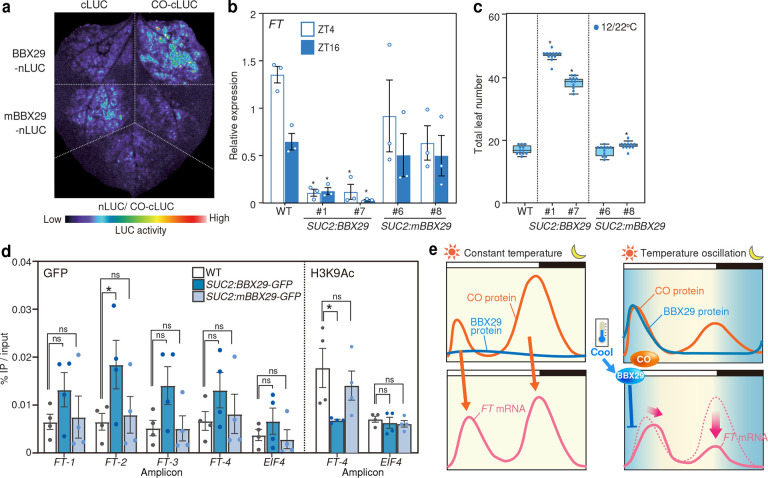
BBX29 directly represses *FT* expression in *CO*-dependent manner **a,** Results from Split-LUC assay in *N. benthamiana* testing the effect of C25S mutation on the protein interaction between BBX29 and CO (left). **b-c**, Phenotypes of *SUC2:BBX29-GFP* plants with or without the B-box mutation. Expression of *FT* (**b**) in plants harvested on ZT4 and ZT16 of 12/22°C. Bars represent the means, and error bars represent plus/minus s. e. m. (*n* = 3). Asterisks indicate significant differences against WT based on Dunnet’s test (*p* < 0.05). **c,** Flowering time results of WT, *SUC2:BBX29-GFP*, and *SUC2:mBBX29-GFP* under 12/22°C. Box plots display the upper and the lower quartiles and the whiskers indicate the 1.5-times interquartile ranges. The horizontal lines in the boxes represent the median. Asterisks indicate significant differences against WT based on Dunnet’s test (*p* < 0.05, *n* = 12). **d**, ChIP-qPCR results testing the binding of BBX29-GFP and mBBX29-GFP at *FT* locus on ZT3 (*n* = 5), and H3K9Ace on ZT5 (*n* = 4). Bars represent the means, and error bars represent plus/minus s. e. m.. Asterisks indicate significant differences against WT controls and ns indicates no significance, based on one-tailed Dunnet’s test (*p* < 0.05). **e,** A model for *FT* regulation under LD conditions with temperature fluctuations. Changes in the daily expression of *FT* and its regulator in response to temperature reduction from nighttime to early morning are shown. Under constant temperature conditions at 22°C, the CO protein accumulates both in the morning and evening to activate *FT* expression. When night-to-morning temperature decreases, there is a reduction in CO protein along with an increased amount of *BBX29* on dawn, which represses morning *FT* expression in a *CO*-dependent manner. We showed that cool temperature from night to morning facilitates multiple pathways to suppress *FT* expression throughout the day to avoid precocious flowering.

To test if CO interaction is sufficient for flowering repression by *BBX29*, we introduced amino acid substitution in BBX29 at the 24^th^ Cys to Ser (C24S) in the Zinc-binding regions of the B-box domain to inhibit homo- and heterodimerization between BBX29 and CO while retaining nuclear localization (Fig. 6a, Supplementary Fig. 10e, f). A similar mutation in BBX19 impaired its interaction with CO^54^, and conserved cysteines are known to hold zinc ion, which is essential for the structural integrity of B-box domain^55,56^. To evaluate the role of BBX29-CO complex in the phloem companion cells, *BBX29-GFP* or *mBBX29-GFP* cDNAs were expressed under the control of the *SUCROSE-PROTON SYNPORTER 2* (*SUC2*) promoter^57^. When *BBX29-GFP* or *mBBX29-GFP* were expressed at the similar levels (Supplementary Fig. 10h), the severe repression of *FT* observed in *SUC2:BBX29-GFP* was reverted to the levels comparable to WT in *SUC2:mBBX29*^*C24S*^*-GFP* (Fig. 6b). Accordingly, the late-flowering phenotype of *SUC2:BBX29-GFP* was largely diminished in *SUC2:mBBX29*^*C24S*^*-GFP* (Fig. 6c). Moreover, DNA-binding observed in *SUC2:BBX29-GFP* was completely abolished in *SUC2:mBBX29-GFP* especially at the *FT-2* region (approximately 0.6kb downstream of *Block C* enhancer), where clusters of CO-NF-Y binding element (CCACA)^58,59^ exist (Fig. 5i and 6d). These results illustrate that physical interaction of BBX29 with CO is required to recruit BBX29 to the *FT* promoter to repress *FT* transcription in the morning to regulate flowering.

Previous study showed that BBX31 bridges the interaction between CO and transcriptional co-repressor TOPLESS (TPL) to regulate *FT* expression^36^. Given that BBX29 also contains the EAR motif, a binding site of TPL, and that TPL recruits the histone deacetylase complex to suppress gene expression^60,61^, we tested if BBX29 changes the histone acetylation level changes on *FT* locus. *SUC2:BBX29-GFP* showed significant reduction in the histone H3 lysine-9 acetylation (H3K9Ac) near the TSS, which was reverted to comparable level to WT in *SUC2:mBBX29*^*C24S*^*-GFP* (Fig. 6d). These results suggest that the binding of BBX29-CO protein complex on *FT* enhancers may indirectly mediate the reduction of H3K9Ac at the *FT* TSS, leading to the repression of morning *FT* expression. Taken all together, our results demonstrate that BBX29-CO protein complex integrates thermoperiodic cues to fine-tune flowering time.

## Discussion

Temperature is a major seasonal cue for regulating flowering responses, but how plants decode temperature information from daily and seasonally fluctuating environments to fine-tune flowering responses has been largely unknown. To address this question, we established temperature conditions that recapitulate diel *FT* expression observed in natural long-day conditions and showed that temperature fluctuations act as active environmental cues, and both temperature and time-of-day information (when the temperature was applied) are important to study plants’ temperature responses (Fig. 1). Similar to how adjusting R-to-FR ratio is sufficient to induce bimodal expression of *FT*^10,16^, our simplified 2-step temperature scheme can be easily implemented in lab growth settings, facilitating to investigate how plants respond to the more realistic ambient temperature changes happening in spring.

Using the 2-step temperature conditions, we demonstrated that daily temperature fluctuations regulate *FT* expression at two distinct time windows through separate mechanisms (Fig. 6e). Under constant 22°C conditions, CO activates expression of *FT* both in the morning and evening. Lowering in night-to-morning temperature to 16°C reduces the amount of CO protein towards dusk transcriptionally and posttranslationally, resulting in the reduction of evening *FT* expression (Fig. 2). Further reduction to 12°C triggers a rapid and coordinated response of *BBX29*, including both transcriptional and translational upregulation, followed by stable complex formation with CO in the vasculature throughout the morning (Fig. 3 and 5). This BBX29-CO protein complex is recruited to the *FT* promoter to downregulate its morning expression (Fig. 5 and 6). Since BBX29 protein contains the EAR motif, which interacts with TPL and TPL-RELATED PROTEINS^60^, BBX29-mediated morning *FT* repression may involve the inhibition of CO transcriptional activity and/or the recruitment of histone deacetylase to the *FT* locus. Our previous work reported that *FT* expression occurs in the morning but not in the evening in plants grown outside in April and May, and both morning and evening peaks become noticeable in plants grown in June^10^. The dual regulatory mechanisms described here may explain how the *FT* expression patterns progress through the spring months.

It has been shown that cool temperature reduces the stability and the mobility of FT protein^62–65^. Therefore, plants may primarily repress evening *FT* to minimize FT protein synthesis and transport during cool nighttime while utilizing morning *FT* as a main source of florigen. Apart from the *BBX29-CO* pathway, cool temperature also stabilizes ZEITLUPE, a LOV-containing F-box protein, which interacts with *FT* transcriptional repressor TARGET OF EAT1 to repress morning *FT* expression^26^. Importantly, the reduction in night-to-morning temperature not only represses morning *FT* induction, but also shifts the peak timing of morning *FT* towards daytime (Fig. 2b). Combinational effects of these genetic pathways may restrict both the amount and timing of FT protein synthesis during warm midday, thereby effectively promote flowering under inductive photoperiods.

The predominant effect of cool temperature on evening *FT* expression may be caused by low accumulation of CO through both transcriptional and posttranscriptional regulation (Fig. 2b and 2f–h). At the posttranslational level, the E3 ubiquitin ligase CONSTITUTIVE PHOTOMORPHOGENIC 1 (COP1) likely contributes to CO degradation^6,10^. Previous studies under constant cool temperature without FR supplementation showed that COP1 is stabilized and enhances its interaction with the substrate adaptor HIGH EXPRESSION OF OSMOTICALLY RESPONSIVE GENES 15, delaying its nuclear exclusion during light period and repress evening *FT* expression^66,67^. On the transcriptional side, cool temperature strongly induces the expression of *CO* transcriptional repressor, *CYCLING DOF FACTOR 6*^68^, as confirmed by our RNA-seq (Fig 3a). These mechanisms are likely active under our 2-step temperature conditions as well and may reduce CO accumulation towards dusk.

We characterized *BBX29* and *BBX31* as negative regulators of morning *FT* regulation under cool temperature. Phylogenetic analyses of the plant BBX family indicate that the BBX family underwent structural diversification early in land plant evolution^69^, yet the involvement of *BBX29* and *BBX31* in low temperature-mediated signaling, from non-freezing to ambient cool temperature, seems to be conserved across eudicots^41,70,71^. BBX29 contains a monopartite nuclear-translocation signal at the C-terminal region, and its nuclear accumulation was slightly enhanced under 12/22°C (Supplementary Fig. 10f). This may involve potential interaction through outside of conserved B-box domain, where functional diversification of BBX family occurs^69^. *BBX29* induction was the most evident in vascular tissues, partially regulated by morning-phased clock genes including *CCA1*/*LHY* (Fig. 3e–i, Supplementary Fig. 5e). As the spatial expression of these clock genes is largely unaffected by cold temperature^43^, it remains an open question how *BBX29* induction is spatially regulated.

Aside from *FT* regulation, *BBX29* and *BBX31* regulate flavonoid production in response to UV signals^72,73^. Although our conditions lacked UV light, our RNA-seq analyses revealed enhanced expression of flavonoid biosynthesis genes under 12/22°C (Fig. 4b), implying that cool temperature alone can enhance this pathway. This raises the possibility that *BBX29* and *BBX31* serve as the signal integration points for UV and cool temperature pathways. Flavonol and anthocyanin function as UV/blue-light absorbing compounds to protect chlorophyll, but they also act as antioxidant to scavenge reactive oxygen species during photoinhibition accelerated by low-temperature stress^74^. Supporting this, chilling temperature around 10°C combined with light has been shown to promote flavonoid accumulation and reduce chlorophyll excitation especially against UV/blue wavelength^75^. Thus, *BBX29* and *BBX31* may contribute to the photoprotection and facilitate vegetative growth during early spring.

In this study, we aimed to understand how temperature fluctuations regulate flowering, with a particular focus on the role of night-to-morning temperatures as a seasonal cue. Our results demonstrated that night-to-morning temperature determines flowering time through *FT* regulation, supporting their roles as time-specific thermal inputs. Importantly, the effect of cool nighttime temperature on flowering time was the most apparent when lower temperatures persisted several hours after dawn (Fig. 1j, Supplementary Fig. 3b), which resembles temperature fluctuations under field conditions. A similar time-dependent effects of temperature have been observed in diel starch metabolism, where significant differences between constant and fluctuating temperature conditions emerge after dawn^76^. Given that temperature rises occur in a few hours after sunrise under field conditions^77,78^, the time lag between the light and temperature cycles may be important to accurately estimate the effect of temperature fluctuation on plant development. Interestingly, our data showed that the effect of cool night-to-morning temperatures persists even after temperature rises during the day, as reflected by CO destabilization toward dusk under 12/22°C conditions (Fig. 2f–h). This suggests that plants can retain the effect of cool temperature beyond the exposure period, through a mechanism distinct from vernalization, which requires prolonged cold and leads to stable epigenetic changes^79^. In contrast, the thermoperiodic regulation described here operates on an hourly timescale and enables flexible and reversible adjustment of flowering in response to recent temperature fluctuations.

We propose the concept of thermoperiodic flowering, defined as flowering regulation by the timing and structure of daily temperature cycles. While photoperiodic control of flowering has been extensively characterized, other environmental signals with intrinsic daily rhythms, particularly temperature, have received limited attention. By developing simplified experimental conditions that capture the essence of natural temperature fluctuation, our study offers a tractable procedure to explore how plants sense seasonal transitions. This approach may serve as a valuable entry point for understanding phenological shifts under natural and changing climates.

## Material and Methods:

### Plant materials and growth conditions.

All experiments were performed with the Columbia-0 (Col-0) accession. The *co-101*, *ft-101*^80^, *ft-1*, *ft-1 tsf*^81^, *FT:GUS*^80^, *CO:HA-CO* and *35S:CO-3F6H*^82^, *35S:YFP-BBX30* and *35S:YFP-BBX31*^48^ used in this study are in the Col-0 background. All plants were grown either on soil in standard flats with seedling starter trays (Tokai Agriculture development) or in sterile 1x Murashige and Skoog medium (Nihon Pharmaceutical Co. Ltd.) supplemented with 0.8% [w/v] agar, 100 mg/L Myo-inositol, 0.4 mg/L thiamine hydrochloride, and adjusted to pH 6.3 with 2-(N-Morpholino) ethanesulfonic acid and KOH. A mixture of vermiculite (Nittai Co., Ltd.) and Sakata Seeds Super Mix A (Sakata Seed Corporation) at a ratio of 2:1 was used in the flowering experiments. After seeds were sown onto soil or growth media, they were stratified in 4°C for at least 48 hours before being transferred to growth conditions. Plants were grown in growth chambers (LH-411PFDT-SP, Nippon Medical Instruments Co., Ltd.) with 16 hours light and 8 hours dark periods. The light source was a plant growth LED (Plantflec bulb color, Nippon Medical Instruments Mfg. Co., Ltd.) supplemented by a 730 nm far-red LED (Namoto Trading Co., Ltd.) in order to set the R/FR=0.8~1. Plants were grown under constant 22°C for the first 10 days after dormancy was broken and transferred to temperature conditions from day 11 to 14. The light spectrum was measured by the spectrophotometer (CL-500A, Konica Minolta). The temperature was directly monitored by HOBO Pendant Temperature/Light 64K Data Loggers (Onset). For gradual temperature setting, the temperature was set every 1 hour to obtain as follows (ZT0, 15.5°C; ZT1, 15.9°C; ZT2, 16.0°C; ZT3, 17.1°C; ZT4, 17.5°C; ZT5, 18.5°C; ZT6, 19.5°C; ZT7, 20.4°C; ZT8, 21.3°C; ZT9, 22.2°C; ZT10, 22.2°C; ZT11, 22.0°C; ZT12, 21.0°C; ZT13, 20.2°C; ZT14, 19.6°C; ZT15, 18.8°C; ZT16, 18.1°C; ZT17, 17.4°C; ZT18, 16.8°C; ZT19, 16.3°C; ZT20, 15.8°C; ZT21, 15.5°C; ZT22, 15.3°C; ZT23, 15.0°C) in order to recreate outside temperature conditions. For 2-step temperature conditions, the temperature was shuttled between low and high on ZT6 and ZT16, unless otherwise described in the text. Flowering time was measured by the number of rosette and cauline leaves on the main stem when inflorescence reached 3 cm high. Flowering time experiments were performed with 10 individual plants at a minimum. All flowering time results in this manuscript are means ± standard errors of means (SEM).

### Climate data analyses

Air temperature measurements were obtained from nearby weather station data https://www.meteoswiss.admin.ch/home/measurement-values.html?param=messnetzflugwetter&station=KLO for Zurich, and http://www-k12.atmos.washington.edu/k12/grayskies/nw_weather.html for Seattle, and https://data.open-power-system-data.org/weather_data/2020-09-16 for the rest of the cities. The geographical location (latitude and longitude) of each city, and the actual time of sunrise and sunset were obtained at https://www.data.jma.go.jp/gmd/cpd/monitor/climatview/frame.php and https://eco.mtk.nao.ac.jp/cgi-bin/koyomi/koyomix.cgi. Nine-year data (2012 to 2020) for Seattle and Zurich, and eight-year data (2012 to 2019) for the rest of the cities were used for the analyses.

### Plasmid construction

To create reporter-fused *CO* and *BBX29* genes driven by their own promoters, approximately 2.6 kb upstream regions of the promoters were amplified from wild-type plants using the following primers; (sequences underlined are necessary for MfeI digestion) 5′-gtatagaaaagttggctccgCTGTTTTGATCATGCAAC -3′ and ttttgtacaaacttgggtcgAATAACTCAGATGTAGTAAGTTTG for *CO*, and 5′-agctCAATTGTTTGTAGAGCGACGCTGAAG-3′ and 5′-agctCAATTGCTCTAGATATTATCTCGCACAACCAC-3′ for *BBX29*. The amplified promoter sequences and coding sequences were cloned into EcoRI-digested pENTR 5′-TOPO vector (Life Technologies) to generate pENTR5′ *pCO* or *pBBX29*, respectively. For luciferase-fused contracts, the coding sequence of firefly luciferase (*LUC*) gene was divided by N-terminal 415 amino acids (designated as nLUC) and C-terminal 459 amino acids (designated as cLUC), then cloned into pENTR-1A (Life technologies) that contains multiple cloning site. Full length of *LUC* was also cloned into pENTR-1A to generate pENTR1A (LUC-nosT)^83^. The coding regions of *CO* and *BBX29* were amplified from cDNA derived from wild-type plants using the following primers; (sequences underlined are necessary for restriction enzymatic digestion) 5′-atgcGTCGACATGTTGAAACAAGAGAG-3′ and 5′-atgcGAATTCGAATGAAGGAACAATCCC -3′for *CO*, 5′-agctGAGCTCggATGGGGAAGAAGTGTGATTTATG-3′5′-agctGGATCCcAACAACAACCGTTGATTTAAACGC-3′for *BBX28*, 5′-acgtGAGCTCGGATGGGGAAGAAGAAGTGCGAG-3′ and 5′-acgtGGATCCcATAAAACGAAGACGACGATG-3′for *BBX29*, 5′-acgtGAGCTCggATGTGTAGAGGCTTGAATAATG-3′and 5′-acgtGGATCCcGAGAAAAACAAACGGAACCTC-3′for *BBX31*, and 5′-acgtGAGCTCggATGGTGAGCTTTTGCGAGC-3′and 5′-acgtGGATCCcAACGTTGTCGTTTTCAGCC-3′for *BBX32*, and then cloned into one of luciferase-containing vectors described above. The resultant plasmids were introduced to R4pGWB501 vector^84^ by using Gateway LR clonase II (Life technologies) to generate *pBBX29:BBX29-LUC, pCO:CO-LUC, pBBX29:BBX29-nLUC*, *pBBX29:BBX29-cLUC*, and *pCO:CO-nLUC*, respectively. *pBBX29:GUS* is created by multi-fragment LR reaction (Life technologies) among pENTR5′ *pBBX29*, *pENTR-GUS* (Life technologies), and R4pGWB501 vector^84^.

To introduce amino acid mutation in B-Box domain of *BBX29-GFP*, coding sequence of *BBX29* fused to EGFP was cloned into pENTR-1A, creating *pENTR BBX29-EGFP*. PCR-based mutagenesis was then introduced to *pENTR BBX29-GFP* by using primers 5′-AGTTTATCTTGGGATTGTGACGGTAAA-3′ and 5′-ATCCCAAGATAAACTCGCTTGATCTGA-3′ to create *pENTR mBBX29-EGFP*. Resultant plasmids were then introduced into pB7WG2 vector^85^ or *pH7WG2-SUC2*pro vector^86^ to create either *35S*- or *SUC2*-driven constructs, respectively.

To generate overexpression lines of GroupV *BBX* family genes, the coding regions of *BBX* genes were amplified by using following primers; 5′-agctGAGCTCggATGGGGAAGAAGTGTGATTTATG-3′5′-agctGGATCCcAACAACAACCGTTGATTTAAACGC-3′for *BBX28*, 5′-acgtGAGCTCggATGGTGAGCTTTTGCGAGC-3′and 5′-acgtGGATCCcAACGTTGTCGTTTTCAGCC-3′for *BBX32*, and then cloned into reporter-containing pENTR vectors. The resultant plasmids were then introduced to pK7WGB2 vector^85^to create 35S-driven GroupV *BBX* family genes.

To generate loss-of-function mutants of *BBX29* and *BBX31* by CIRISPR/Cas9, two single gRNAs targeting B1 domain of BBX29 and BBX31 were designed as follows; 5′-AGATCAAGCGAGTTTATGTT-3′ and 5′-GATTGTGACGGTAAAGTTCA-3′ for BBX29 and 5′-TCGGTCTCGTGCAGAGACTC-3′ and 5′-CGCCTCCGTGTTCTGTGAAG-3′ for BBX31. The sgRNAs fused to the Arabidopsis tRNA-Gly gene were amplified using pGTR (Addgene, plasmid 63143) as PCR template. The primers for the PCR are 5’-tcgaagtagtgattgAGATCAAGCGAGTTTATGTTgttttagagctagaaatagcaagttaaaat-3’, 5’-TGAACTTTACCGTCACAATCtgcaccagccgggaa-3’ for BBX29, and 5’-tcgaagtagtgattgTCGGTCTCGTGCAGAGACTCgttttagagctagaaatagcaagttaaaat-3’ and 5’-CTTCACAGAACACGGAGGCGtgcaccagccgggaa-3’ for BBX31.The resulting PCR products harboring the two sgRNA sequences were cloned into the pKI1.1R vector (Addgene, plasmid 85808) digested with AarI using NEBuilder HiFi enzyme (NEB). All vectors used in this study were introduced in *Arabidopsis* Col-0 by floral-dip method using *Agrobacterium* strain GV3101. Transgenic plants were selected on culture media containing appropriate antibiotics, and T3 homozygous plants were selected for further experiments. For *bbx29 bbx31* loss-of-function mutants created by CRISPR/Cas9, T3 transgenic plants without the CRISPR/Cas9 construct were selected for the analyses.

### RNA preparation and gene expression analyses.

For full time-course analyses diel gene expression analyses, 14-day old seedlings grown on MS media were harvested every 3 hours starting from ZT1 and used for RNA extraction by using FavorPrep Plant Total RNA Mini Kit (Chiyoda Science) according to the manufactures’ instruction. Mesophyll and vasculature isolation was performed as described previously^44^ by using 7-day-old plants harvested on ZT1 and subjected to RNA extraction by using RNeasy Plant Mini Kit (Qiagen). For all the experiments, plants were grown under 22/22°C, 16/22°C, or 12/22°C conditions for 3 days prior to the harvesting unless otherwise stated. RNA samples were reverse-transcribed using Prime Script RT reagent Kit with gDNA Eraser (Takara), and subjected to real-time gene expression analyses with CFX96 Real-time Detection System (BioRad). Primers used for qPCR were listed in Supplemental Table 2. All expression results were normalized using averages of *IPP2* and *PP2A* values, and represented as relative values to the geometric mean value from WT, or 2-step temperature condition (16/22°C, ZT6–16), unless otherwise stated. Data was presented at least from three independent biological replicates.

### Histochemical GUS staining

Seven- or 14-day old seedlings were sampled on ZT4 and fixed in 90 % acetone for 10 min on ice, rinsed twice with 100 mM NaPO4, then vacuum-infiltrated at 37°C for 4hrs (overnight for *FT:GUS*) in GUS staining solution [100 mM NaPO4, 10 mM EDTA, 0.5 mM K_4_Fe(CN)_6_, 0.5 mM K_3_Fe(CN)_6_, 0.1 % Triton, 1 mM X-Gluc]. Chlorophylls in the tissue were removed by incubation in 70% (v/v) ethanol, followed by overnight incubation with chloral hydrate decolorizing solution [80 g chloral hydrate, 20 mL glycerol, 10 mL Elix water]. Samples were mounted with 50% glycerol and observed under a stereomicroscope [MZ FLIII, Leica].

### Immunoblot analysis and protein quantification.

For analyzing daily expression profiles of CO and BBX29 proteins, 14-day-old seedlings grown on agar media were used. Total proteins were extracted using extraction buffer [50 mM Na-phosphate pH7.4, 100 mM NaCl, 10% (v/v) glycerol, 5 mM EDTA, 1 mM DTT, 1% NP-40, 0.5% SDS, 0.5% sodium deoxycholate, 50 μM MG-132, 2 mM NaVO_4_, 2 mM NaF, and Pierce protease inhibitor tablets-EDTA free (Thermo)], and nuclei samples were prepared using CelLytic Plant Nuclei Isolation/Extraction Kit (Sigma-Aldrich) as described previously^87^. To detect proteins, total protein extract or nuclear extract, were resolved in 13% SDS-PAGE gels and transferred to PVDF membranes (Millipore). Histone H3 proteins were used for internal loading controls. HA-CO, BBX29-GFP, and H3A proteins were detected using anti-HA (3F10, Roche), anti-GFP (GF200, Nakarai Tesque, Kyoto, Japan), and anti-histone H3 (MABI0301, MBL, Nagoya, Aichi, Japan) antibodies. HRP-conjugated goat anti-mouse antibody (MBL330, MBL) was used as a secondary antibody. For protein detection, immunoreactive proteins on immunoblotted membranes were visualized by LAS-3000 (GE) with SuperSignal West Pico PLUS or Atto Chemiluminescence Substrate Solution (Thermo), or ECL Select Western Blotting Detection Reagent (Cytiva), depending on the signal intensities. The image was used for quantification with the Image Studio Lite (LI-COR, Lincoln, LE, USA). Relative protein abundance was normalized against histone H3, followed by the normalization against a geometric mean of 22/22°C condition. Data from at least three biological replicates were presented.

### Chromatin immunoprecipitation and qPCR

ChIP assay for BBX29-GFP and H3K9ac was performed with twelve-day -old seedlings as described previously^88,89^. For BBX29-GFP ChIP, samples were homogenized and cross-linked in the extraction buffer containing formaldehyde [0.4 M sucrose, 10 mM HEPES (pH 8.0), 2 mM EDTA, 5 mM β-mercaptoethanol, EDTA-free protease inhibitor tablet (Thermo Fisher Scientific), 1% formaldehyde] for 10 min at 4 °C, followed by quenching with a total concentration of 200 mM glycine for 5 min. Cross-linked samples were filtered twice through Miracloth. Chromatin was isolated and sonicated with Bioruptor Pico (Diagenode, Denville, NJ, USA) 5 times (30 sec on/30 sec off cycles and high-power output). The sonicated chromatin was precipitated with anti-GFP antibody (SAB4301138, Sigma-Aldrich). For the histone ChIP, approximately 0.6 g of seedlings were harvested into PBS (137 mM NaCl, 2.7 mM KCl, 10 mM Na_2_HPO_4_, 2 mM KH_2_PO_4_, pH 7.4) and fixed in 1% (v/v) formaldehyde under vacuum (three cycles) for 15 min. Fixation was quenched with 0.125 M glycine for 5 min, and seedlings were rinsed with cold PBS and frozen in liquid nitrogen. Frozen tissue was ground to a fine powder and suspended in Nuclei Extraction Buffer (100 mM MOPS, pH 8.0, 10 mM MgCl_2_, 0.25 M sucrose, 0.5% dextran T-40, 2.5% Ficoll 400, 9.36 μL/mL β-mercaptoethanol, 10 μL/mL protease inhibitors). After filtration and centrifugation, chromatin pellets were lysed in SDS buffer (10 mM EDTA, 50 mM Tris-HCl, pH 8.0, 1% SDS), diluted in ChIP buffer (16.7 mM Tris-HCl, pH 8.0, 1.2 mM EDTA, 167 mM NaCl, 1.1% Triton X-100, 0.01% SDS), and sonicated to obtain DNA fragments of 200–700 bp. After debris removal and pre-clearing with Dynabeads^™^ Protein A, input samples were collected and ChIP was performed overnight at 4°C with antibodies (H3K9ac, ab10812; Abcam). The immunoprecipitated DNA after reverse-crosslinking was purified using PureLink PCR Purification Kit (Life technologies). qPCR was performed with SsoAdvanced Universal SYBR Green Supermix (Biorad). Primers and PCR conditions were listed in Supplemental Table 2.

### RNA sequencing analysis

To prepare samples, 14-day-old WT plants with 3-day entrainment of 22/22°C or 12/22°C were harvested at ZT0 and ZT4. The extracted total RNAs were converted to 3′ digital gene expression RNA-seq libraries based on Lasy-Seq protocols^90^. Paired-end 150 bp sequencing was conducted using the HiSeq system (Illumina). The obtained sequences were filtered using Trimmomatic v0.39 with option “LEADING:24 TRAILING:24 SLIDINGWINDOW:30:20 AVGQUAL:20 MINLEN:60”^91^. The filtered reads were mapped to *A. thaliana* cDNA references downloaded from the TAIR website and counted by RSEM v1.3.1 with bowtie2 v2.4.2^92,93^. The edgeR analysis was performed to detect cold induced genes (log FC > 1 and Log CPM > 1) in ZT0 and ZT4. The transcription factors were extracted based on GO slim information (GO:0003700, DNA-binding transcription factor activity) provided by the TAIR website.

### Tissue clearing and confocal microscopy

Tissue clearing was conducted following Kurihara et al. with minor modifications^94^. The first and second true leaf blades of 2-week-old *BBX29:BBX29-GFP* grown under 12/22°C and 22/22°C were cut and immersed in 1mL 4% paraformaldehyde in a microtube. Leaves in the tube were placed in a desiccator with the pressure adjusted to 690 mmHg for 2 min, and pressure was slowly released not to abrupt samples. Pressure in the desiccator was adjusted to 690 mmHg again for an hour and released pressure slowly. Paraformaldehyde in the tube was carefully removed by pipetting. After washing leaves with 1xPBS twice, ClearSee (FUJIFILM Wako Pure Chemical Co.) was added to the tube. Samples in ClearSee were placed in a desiccator adjusted to 690 mmHg for 2 min, and pressure was released slowly. Subsequently, the pressure was adjusted to 690 mmHg for an hour and released slowly. Leaf samples in the tube were kept in a covered box, and ClearSee solution was replaced with a new one until the tissues became transparent.

Confocal images were taken using LSM780-DUO-NLO (Zeiss) equipped with 32-channel spectral GaAsP detector and a Plan-Apochromat 10×/0.45 and 20×0.8 objective lens. To extract GFP fluorescence and remove autofluorescence, fluorescent spectra of GFP and autofluorescence excited with a 488 nm were used as references. DAPI channel was Ex 405 nm and Em 410–585 nm.

### Detection of bioluminescence

For the quantification of luciferase bioluminescence, two-week-old plants were sprayed with 2.5 mM D-luciferin (Biosynth) one day before imaging, and used for photon counting by a photomultiplier-tube-based bioluminescence monitoring system32^95^.To perform *in vivo* split-LUC assay between BBX29 and CO, *Arabidopsis* stable transgenic lines expressing *BBX29:BBX29-n/cLUC* and *CO:CO-cLUC* were independently generated, and then crossed to establish F3 generations for the measurement. Same constructs without coding sequence of BBX29/CO were used for the negative control. In each measurement, five or six seedlings were subjected to photon counting. For the quantitative analyses of *CO:CO-LUC* and *BBX29:BBX29-LUC*, single plant per each measurement was used for photon counting. Average data from approximately five to ten measurements was presented.

For luminescence imaging, luciferin was sprayed right before the imaging. Luminescence was observed using a Multi-function *in vivo* imager, controlled by Metamorph software (Molecular Devices, San Jose, California, USA).

### Transient assay with *N. benthamiana*

To observe the subcellular localization of BBX29 and mBBX29, approximately 30-day-old *N. benthamiana* plants were used for agroinfiltration. The *Agrobacterium* strain GV3130 containing plasmids of interest was grown to the stationary phase. Bacterial cells were harvested by centrifugation and resuspended to OD_600_ of 0.2 in MES buffer [10 mM MgCl2, 10 mM MES pH 5.6, 150 μM acetosyringone (Sigma-Aldrich)]. After 4 h of incubation at room temperature in MES buffer, *Agrobacterium* solution was infiltrated into the abaxial air spaces of leaf tissue using 1-mL syringes. Two to three days after infiltration, images of the tissue were analyzed with a confocal laser scanning microscope (TCS SP8; Leica Microsystems). Approximately 50 to 100 cells that express H2B-RFP were observed in each combination. H2B-RFP was used for the nuclear marker.

To perform split-LUC assay among BBX29 and CO in *N. benthamiana* leaves, *Agrobacterium* containing 35S-driven fusion constructs in which nLUC or cLUC was fused BBX29 and CO were mixed at a same amount and co-infiltrated. The same constructs that express only nLUC or cLUC were used as a control.

### Statistical analysis

Statistical analyses were done using R (v4.0.5; R core team) and GraphPad Prism software (v10 for windows, GraphpaPad software). The statistical significance in pairwise comparison was determined using Welch’s T-test or Student’s T-test. Multiple omparisons were determined using either Dunnet’s test or Tukey’s honestly significant difference (HSD) test as described in the legends.

## Supplementary Files

This is a list of supplementary les associated with this preprint. Click to download.

- SupplementaryTable1.xlsx
- SupplementaryTable2.xlsx
- KubotaetalSI.pdf

## Data Availability

All data are available in the main text or the Supplementary Materials. The raw sequence data (PRJDB35651) were deposited in the NCBI Sequence Read Archive.
